# Mutation patterns of mtDNA: Empirical inferences for the coding region

**DOI:** 10.1186/1471-2148-8-167

**Published:** 2008-06-02

**Authors:** Cristina Santos, Rafael Montiel, Adriana Arruda, Luis Alvarez, Maria Pilar Aluja, Manuela Lima

**Affiliations:** 1 Center for Research in Natural Resources (CIRN), University of the Azores, 9500 Ponta Delgada, S. Miguel, Azores, Portugal; 2 Biological Anthropology Unit, Department BABVE, Faculty of Sciences, Autonomous University of Barcelona, 08193 Bellaterra (Barcelona), Spain

## Abstract

**Background:**

Human mitochondrial DNA (mtDNA) has been extensively used in population and evolutionary genetics studies. Thus, a valid estimate of human mtDNA evolutionary rate is important in many research fields. The small number of estimations performed for the coding region of the molecule, showed important differences between phylogenetic and empirical approaches. We analyzed a portion of the coding region of mtDNA (*tRNA*^*Leu*^, *ND1 *and *tRNA*^*Ile *^genes), using individuals belonging to extended families from the Azores Islands (Portugal) with the main aim of providing empirical estimations of the mutation rate of the coding region of mtDNA under different assumptions, and hence to better understand the mtDNA evolutionary process.

**Results:**

Heteroplasmy was detected in 6.5% (3/46) of the families analyzed. In all of the families the presence of mtDNA heteroplasmy resulted from three new point mutations, and no cases of insertions or deletions were identified. Major differences were found in the proportion and type of heteroplasmy found in the genes studied when compared to those obtained in a previous report for the D-loop. Our empirical estimation of mtDNA coding region mutation rate, calculated taking into account the sex of individuals carrying new mutations, the probability of intra-individual fixation of mutations present in heteroplasmy and, to the possible extent, the effect of selection, is similar to that obtained using phylogenetic approaches.

**Conclusion:**

Based on our results, the discrepancy previously reported between the human mtDNA coding region mutation rates observed along evolutionary timescales and estimations obtained using family pedigrees can be resolved when correcting for the previously cited factors.

## Background

There are several reasons why the mitochondrial genome (mtDNA) of humans as well as of other mammalians has been considered to be useful in population genetics, phylogeographic, and phylogenetic studies. Besides the usually invoked mtDNA characteristics (high copy number per cell, compact organization and maternal transmission), mtDNA has been widely used because it provides easy access to an orthologous set of genes with little or no recombination and rapid evolution [1]. Moreover, from a theoretical perspective, it has been accepted for a long time that mtDNA haplotype frequencies are controlled primarily by migration and genetic drift and that most of the variation within a species is selectively neutral [1]. However, more recent reports sustain the hypothesis that mtDNA frequency variation is due to natural selection [2-4].

Given the importance of mitochondrial function, it is not straightforward to assume *a priori *that mtDNA evolves as a strictly neutral marker. Changes in the mtDNA sequence can have substantial impacts on the fitness of the organelle/cell (within individuals) and on the fitness of the individual host organism. Deviations from a strictly neutral model of evolution have been found in a variety of organisms [5-9]. Ballard and Rand [1], in a revision, gave three main reasons why it is reasonable to predict that mtDNA variation may be under strong selection: i) Mitochondrion is the powerhouse of the cell and, in most organisms, a reduction in ATP production is expected to reduce fecundity. In humans, a reduction in the efficiency of ATP production is known to be highly deleterious or lethal in the extreme case; ii) Proteins from mtDNA interact with those imported from the nuclear genome to form four of the five complexes of the electron transport chain; and iii) The assumption of total absence of recombination in mtDNA means that each genome has a single genealogical history and all genes will share that history. Any evolutionary force acting at any one site will equally affect the history of the whole molecule. Thus, the fixation of an advantageous mutation by selection, for example, will cause the fixation of all other polymorphisms by a process known as "genetic hitchhiking" [10]. Even the quickly evolving non-coding mtDNA region (D-loop) cannot be assumed to have neutral allele frequencies: It is linked to the rest of the genome (where selection has been documented) and conserved motifs within this region exhibit variation that affects mitochondrial transcription and replication in significant ways [11]. Alternatively, polymorphism within a mitochondrial genome may be depressed through selection against linked deleterious mutations, a process known as "background selection" [12-14].

Besides the significance of mtDNA selection *per se*, the assessment of the impact of selection in mtDNA is crucial in the establishment of mtDNA evolutionary rate, as was previously demonstrated by Denver et al. [7]. In a strict neutral model of evolution, the substitution rate depends only on the mutation rate per individual; however, if there was any, even if slight, effect of selection this would not be true. According to Ohta [15,16] very slightly deleterious mutants are effectively selected against in populations large enough; however these same mutants should be governed by random drift in small populations behaving as selectively neutral. Thus, small populations may accumulate deleterious mitochondrial mutations at an increased rate [17].

Human mtDNA substitution rate has been estimated using mainly phylogenetic [18-29] and empirical methods [30-37]. Estimations for the D-loop, based on phylogenetic approaches, range from 0.0575 mutations/site/Myr [20] to 0.2860 mutations/site/Myr [21], whereas estimations using an empirical methodology range from 0 mutations/site/Myr [33,35] to 2.5 mutations/site/Myr [32]. On what concerns the coding region, the few estimations performed so far showed that the substitution rate of the coding region is ~10 times lower than that reported for the D-loop; furthermore, and as observed for the D-loop, important differences between phylogenetic and empirical estimations have been pinpointed [31]. Mishmar et al. [2] applying a phylogenetic approach to complete sequences of the coding mtDNA region obtained a substitution rate of 0.0126 mutations/site/Myr, whereas Howell et al. [31], using an empirical estimation for the coding region of mtDNA, report a value of 0.075 mutations/site/Myr.

Since the first empirical D-loop estimation of mtDNA evolutionary rate by Howell et al. [30] an intense debate about the causes of the discrepancies between phylogenetic and empirical rates has taken place [9,31,34,36-40]. Such discrepancy has been attributed to distinct causes, namely: to differences in the rate of mutation at different mtDNA positions; to the effect of selection and genetic drift; to the occurrence of somatic mutations; to the unintended sequencing of nuclear mitochondrial pseudogenes; and to the leakage of paternal mtDNA and recombination. Moreover Ho et al. [9,41] described an acceleration of the rate of substitution at evolutionarily short timescales and among other factors, the authors attributed this acceleration to purifying selection acting on mtDNA [9,41]. However, Emerson [42] reanalyzing the data of Ho et al. [9] suggested that it would seem that the time-scale upon which the pedigree rate converges to the evolutionary rate is very much shorter than the timescale that Ho et al. [9] have focused on, and it is debatable whether this convergence would follow an exponential distribution, or if such a pattern existed, whether it could not be equally explained by coalescent effects.

Recently, we reported on the mtDNA mutation rate of the D-loop [37], using an empirical approach. Our results supported the conclusion that the discrepancy between phylogenetic and pedigree derived rates cannot be attributed neither to the inclusion of somatic mutations in calculations, nor to the use of families with mtDNA disease, or even to paternal contribution of mtDNA. Moreover, the discrepancy cannot be justified by the fact that mutations observed in families occurred preferentially in hypervariable sites. Santos et al. [37] advanced two additional factors that must be taken into account: the gender of individuals carrying germinal mutations (mutations carried exclusively by men will never be passed to the next generation) and "the weight" of heteroplasmic germinal mutations (for mutations in mtDNA to reach polymorphism levels in the population – and eventually become fixed – it is first necessary that they pass from an heteroplasmic to an homoplasmic state, at the individual level, and this will be dictated by the initial levels of heteroplasmy).

To date there are only two studies that deal with the empirical mutation rate estimation of coding mtDNA region [31,35]. Moreover, the discussion of the effect of selection in the fate of new arising mutations in coding and non-coding portions of mtDNA has not been fully addressed. In this work we present the results of the analysis of a portion of the coding region of mtDNA, using individuals belonging to extended families from the Azores Islands (Portugal). The main aims are: a) to provide empirical estimations of the mutation rate of the coding region of mtDNA under different assumptions, and b) to better understand the mtDNA evolutionary process, including the factors that control the levels and progress of mtDNA heteroplasmy until the intraindividual fixation of new arising mutations.

Heteroplasmy was detected in 6.5% of the families analyzed. In all of the families the presence of mtDNA heteroplasmy resulted from three new point mutations, and no cases of insertions or deletions were identified. Our empirical estimation of mtDNA coding region mutation rate, calculated taking into account several factors is similar to that obtained using phylogenetic approaches.

## Results

### *tRNA*^*Leu*^, *ND1 *and *tRNA*^*Ile *^genes sequence analysis in Azorean pedigrees

**Figure 1:**
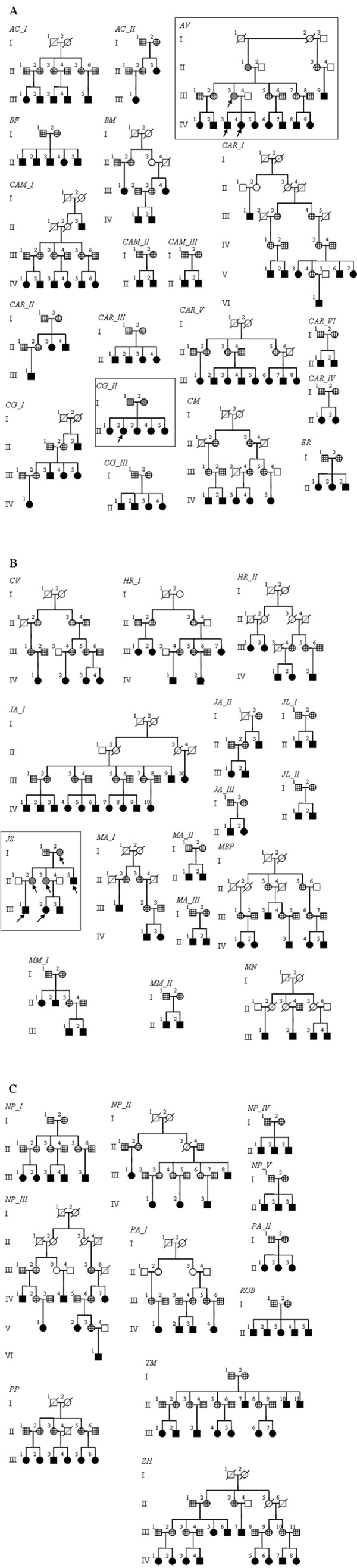
**Forty-six pedigrees relating sampled individuals.** Individuals represented by filled black squares and circles were analyzed in the first step; those represented by half tone squares and circles were analyzed after detection of heteroplasmy in individuals analyzed in the first step; samples of individuals represented by grille squares and circles were available but were not analyzed; samples of individuals represented by white squares and circles were not available. In all cases heteroplasmy is signaled with arrows.

The mtDNA coding region between nucleotide positions 3230 and 4331 (numbering according to Andrews et al. [43]), was fully sequenced in 208 individuals from the most recent generation and in 7 of their relatives (4 mothers, 2 grand-mothers and 1 father) (Fig. 1). The sequences of *tRNA*^*Leu*^, *ND1 *and *tRNA*^*Ile *^obtained are available as additional file (see Additional file 1).

From the 208 individuals first analyzed, six appear to present mtDNA heteroplasmy in the fragment of the coding region that was analyzed. The six individuals presenting heteroplasmy belong to three distinct families, which will be further described. Thus, 6.5% (3/46) of the families present mtDNA heteroplasmy produced by mutations in the region analyzed. In all of the cases, heteroplasmy was produced by a point mutation and no cases of insertions or deletions have been detected.

### Description and segregation of heteroplasmy in *tRNA*^*Leu*^, *ND1 *and tRNA^*Ile *^genes

In Table 1, the individuals carrying heteroplasmy, the heteroplasmic positions and the levels of heteroplasmy (ascertained by the height of peaks in the electropherogram and by clone counting) are presented for each family. In Table 2, the type and functional implication of mutation, and the nucleotide in the homoplasmic individuals of the family and in reference sequences [28,44], is presented for each heteroplasmic position. Moreover, the number of hits, based on the mtDNA phylogeny [45] is also presented.

**Table 1:** For each family analyzed, code (according to figure 1), sex and proportion of variants in individuals presenting heteroplasmy.

Family	Individual	Sex	Position	Proportion height peaks Forward Primer	Proportion height peaks Reverse Primer	Mean proportion height peaks	Proportion clone number (N)
AV	IV3	M	3260 A>G	84.6%A : 15.4%G	85.7%A : 14.3%G	85.15%A : 14.85%G	-
	IV4	F	3260 A>G	82.6%A : 17.4%G	77.8%A : 22.2%G	80.20%A : 19.80%G	81.8%A : 18.2%G (22)
	(III3)	F	3260 A>G	95.6%A : 4.4%G	96.2%A : 3.8%G	95.90%A : 4.10%G	-

CG_II	II2	F	3396 T>C	87.5%T : 12.5%C	82.4%T : 17.6%C	84.95%T : 15.05%C	-

JS	III1	M	3602 A>G	87.7%A : 12.3%G	88.4%A : 11.6%G	88.05%A : 11.95%G	-
	III2	F	3602 A>G	92.3%A : 7.7%G	96.0%A : 4.0%G	94.15%A : 5.85%G	98.0%A : 2.0%G (50)
	II5	M	3602 A>G	64.7%A : 35.3%G	66.7%A : 33.3%G	65.70%A : 34.30%G	-
	(II2)	F	3602 A>G	81.8%A : 18.2%G	84.2%A : 15.8%G	83.00%A : 17.00%G	-
	(II3)	F	3602 A>G	82.8%A : 17.2%G	85.7%A : 14.3%G	84.25%A : 15.75%G	-
	(I2)	F	3602 A>G	88.2%A : 11.8%G	89.3%A : 10.7%G	88.75%A : 11.25%G	-

**Table 2:** Characterization of positions presenting heteroplasmy.

Family	Position	Type of mutation and Implication	Homoplasmic individuals of the family/Reference^a^/Chimpanzee^b^	Germline	Number of hits^c^	Population Database frequency
AV	3260 A>G	Substitution in the *tRNA*^*Leu *^associated with Myopaty and Cardiomiopaty	A/A/A	Yes	0	100% A 0% G
CG_II	3396 T>C	Synonymous substitution in *ND1*	T/T/C	No?	4	99.73% T 0.27% C
JS	3602 A>G	Nonsynonymous substitution in *ND1 *that implies an Asparagine to Serine amino acid change.	A/A/A	Yes	0	100% A 0% G

a. Ingman et al. [28], GenBank accession number NC_001807

b. Arnason et al. [44], GenBank accession number X93335

c. Based on the mtDNA phylogeny available on Ruiz-Pesini et al. [45]

Families showing point heteroplasmy can be subdivided into:

1) One family in which only one individual presented heteroplasmy produced by a point mutation:

*- Family CG_II *(Fig. 1): in the first step of the analysis we sequenced the *tRNA*^*Leu*^, *ND1 *and *tRNA*^*Ile *^region of five maternally related individuals (5 women) from which, one (II2) displayed low levels of heteroplasmy (84.95%T:15.05%C) as the result of a T->C mutation in position 3396 (Table 1). This mutation, although in a coding region, is a synonymous mutation since it does not imply an aminoacid change and the mitotype C^(3396) ^was detected, although at low frequency, at the population level (Table 2). In this family the reported heteroplasmy may have resulted from a somatic mutation that occurred in the individual herself, or alternatively, it may have resulted from germline mutations that occurred in the oocyte from which the heteroplasmic individual developed. Thus, it is not clear if this mutation can be transmitted to the next generation.

2) Two families in which various individuals displayed heteroplasmy produced by a point mutation:

*- Family AV *(Fig. 1): we first sequenced the 3230–4331 region in ten maternally related individuals (5 men and 5 women). From these, one man (IV3) and one woman (IV4) showed low levels of heteroplasmy (85.15%A:14.85%G and 80.20%A:19.80%G respectively) as a consequence of a point mutation in position 3260 of the *tRNA*^*Leu*(UUR) ^(Table 1). The subsequent analysis of their mother (III3) and grandmother (II1) showed the presence of very low levels of heteroplasmy in the mother (95.90%A:4.10%G) and the absence of heteroplasmy in the grandmother. Thus, it is likely that the transition from A to G in position 3260 occurred in individual III3 and was transmitted to individuals IV3 and IV4.

*- Family JS *(Fig. 1): The 3230–4331 region was first sequenced in four maternally related individuals (3 men and 1 woman). From these, one man (II5) showed medium levels of heteroplasmy (65.70%A:34.30%G), whereas another man (III1) and one woman (III2) showed low levels of heteroplasmy (88.05%A:11.95%G and 94.15%A:5.85%G respectively) (Table 1). The heteroplasmy resulted from a point mutation in position 3602 of the *ND1 *gene (Table 1). This mutation is a non-synonymous mutation that changes the aminoacid residue 99 of the *ND1 *sub-unit from asparagine to serine. The subsequent analysis of their maternal ancestors (I2, II2 and II3) showed the presence of low levels of heteroplasmy in all of them (G^(3602) ^mitotype in a frequency inferior to 17%). Thus, it was not possible to identify directly the individual where the mutation arose and, by consequence, the ancestral state for this position in this family. If we take into account that this position appears to be invariant at the population level (100% A) (Table 2) and that the state in the reference sequences [28,44] is an adenine, we can hypothesize that this mutation could represent an A to G change at position 3602. As the conservation index of this mutation is 100%, we may consider that it may have some functional consequence and that is not neutral. However, at this point we cannot predict if, this mutation will be deleterious or beneficial.

### Overall Analysis of Mutations: mutation pattern and mutation rate in *tRNA*^*Leu*^, *ND1 *and *tRNA*^*Ile *^genes

We detected three transitions that gave rise to heteroplasmy in three distinct positions. One can be somatic or germinal and two can be considered germinal (Table 2). One mutation was detected in the *tRNA*^*Leu *^and the other two were found in the *ND1 *gene. All the mutations take place at sites that present a very low number of hits in the mtDNA phylogeny, a high conservation index, and that don't appear to be polymorphic in human populations.

In 311 mtDNA transmissions we detected three substitutions in the 1102 bp analyzed (positions 3230 to 4331: *tRNA*^*Leu*^, *ND1 *and *tRNA*^*Ile *^genes), which implies that 0.0096 new mutations arise (at a detectable level) in each generation, corresponding to 8.7×10^-6 ^mutations/site/generation. If we employ the same definition of mutation used by other authors (for example Howell et al. [31]), only mutations for which there is evidence that they are germinal should be considered (see Table 2). This implies that the mutation rate would be reduced to 2 mutations in 311 mtDNA transmissions, that is, 0.0064 mutations/generation for the entire region or 5.8×10^-6 ^mutations/site/generation (95% CI: 0–13.61×10^-6^). This last value is ~4 times higher than that reported by Howell et al. [31] for the coding region (1.5×10^-6 ^mutations/site/generation) and ~3 times lower than that reported by Santos et al. [37] for the D-loop using the same criteria of including only germinal mutations. Assuming that the generational time is 25 years, the mutation rate in the coding fragment analyzed would be 0.2334 mutations/site/Myr (95% CI: 0–0.544). As in the study of Howell et al. [31], this empirical estimation of mutation rate for the coding region is much higher than estimations obtained by phylogenetic methods (Table 3).

**Table 3:** Substitution rate of Human mtDNA obtained in this study and in other studies using different methods (for a more extensive list see Santos et al [37]).

Reference	Region	Method	Human-chimpanzee divergence time (Myr)	Substitution rate as expressed by authors	Standardized substitution rate mutations/site/Myr^a^
Horai et al. [27]	Synonymous positions	Phylogenetic	4.9	3.89 × 10^-8 ^mut/site/year	0.0389
Kivisild et al. [29]	4212 synonymous positions	Phylogenetic	6	3.5 × 10^-8 ^transversions/site/year	0.035
Kivisild et al. [29]	Synonymous positions	Phylogenetic	6	2.1 × 10^-9 ^transversions/site/year	0.0021
Kivisild et al. [29]	rRNA positions	Phylogenetic	6	4.1 × 10^-10 ^transversions/site/year	0.00041
Cann et al. [19]	Complete mtDNA	Phylogenetic	-	0.01–0.02 mut/site/Myr	0.01–0.02
Torroni et al. [26]	Complete mtDNA	Phylogenetic	-	0.00022–0.00029 mut/site/10000	0.022–0.029
Brown [18]	Complete mtDNA	Phylogenetic	-	0.005–0.01 mut/site/Myr	0.005–0.01
Vigilant et al. 1991	HVR I and II	Phylogenetic	6	17.3%/Myr	0.0865
Tamura and Nei 1993	HVR I and II	Phylogenetic	4–6 Myr	1.5×10^-7^-2.5×10^-8^/site/year	0.1500–0.0250
Pesole et al. 1992	HVR I and II	Phylogenetic	7.5 Myr	18 ± 10/100 site/Myr	0.1800 ± 0.1
Ho et al. [41]	D-loop	Population (calibration 80 kyr)	-	0.197 (0.115–0.293) mut/site/Myr	0.197 (0.115–0.293)
Santos et al. [37]^(1)^	D-loop	Pedigree	-	3.5219×10^-5^/site/generation	1.4088
Santos et al. [37]^(2)^	D-loop	Pedigree	-	1.9210×10^-5^/site/generation	0.7684
Santos et al. [37]^(4)^	D-loop	Pedigree	-	1.2807×10^-5^/site/generation	0.5123
Santos et al. [37]^(6)^	D-loop	Pedigree	-	4.1878×10^-6^/site/generation	0.1675
Heyer et al. [36]^c^	HVR I and II	Pedigree	-	11.7/site/million generations	0.4680
Sigurðardóttir et al [34]^c^	HVR I and II	Pedigree	-	0.32/site/Myr	0.2529
Parsons et al [32]^(1)^	HVR I and II	Pedigree	-	2.5/site/Myr	1.7957
Howell et al [31]^d (2)^	D-loop	Pedigree	-	9.48×10^-6 ^mut/site/generation	0.3791
Mishmar et al. [2]	Coding region	Phylogenetic	6.5	1.26 × 10^-8 ^mut/site/year	0.0126
Ingman et al. [28]	Coding region	Phylogenetic	5	1.70 × 10^-8 ^mut/site/year	0.017
Howell et al. [31]^(2)^	Coding region^b^	Pedigree	-	1.52 × 10^-6 ^mut/site/generation	0.0609
**Present study^(1)^**	1102 bp Coding region	Pedigree	-	8.75 × 10^-6 ^mut/site/generation	0.3501
**Present study^(2)^**	1102 bp Coding region	Pedigree	-	5.84 × 10^-6 ^mut/site/generation	0.2334
**Present study^(3)^**	1102 bp Coding region	Pedigree	-	8.75 × 10^-6 ^mut/site/generation	0.3501
**Present study^(4)^**	1102 bp Coding region	Pedigree	-	5.84 × 10^-6 ^mut/site/generation	0.2334
**Present study^(5)^**	1102 bp Coding region	Pedigree	-	1.03 × 10^-6 ^mut/site/generation	0.0411
**Present study^(6)^**	1102 bp Coding region	Pedigree	-	5.89 × 10^-7 ^mut/site/generation	0.0236
**Present study^(7)^**	1102 bp Coding region	Pedigree	-	4.39 × 10^-7 ^– 4.97 × 10^-7 ^mut/site/generation	0.0176–0.0199
**Present study^(8)^**	1102 bp Coding region	Pedigree	-	0–5.84 × 10^-8 ^mut/site/generation	0–0.0023

a. Assuming a generational time of 25 years;

b. Assumed 15447 pb but used fragments of coding region; 4 mutations in 170 transmissions.

c. All the mutations considered were in homoplasmy.

d. Value pooling all the family studies performed until 2003 (table 2 in Howell et al. [31])

(1) All the substitutions that were detected were considered for the mutation rate calculation;

(2) Only the substitutions for which there was evidence of a germinal origin were considered;

(3) Only the substitutions present in women were considered;

(4) Only the substitutions present in women for whom there was evidence of a germinal origin were considered;

(5) Only the substitutions present in women that would become fixed at the individual level considering neutrality were considered;

(6) Only the substitutions with a confirmed germinal origin present in women that would become fixed at the individual level considering neutrality were considered;

(7) Only the substitutions present in women that would become fixed at the individual level considering the effect of selection;

(8) Only the substitutions with a confirmed germinal origin present in women that would become fixed at the individual level considering the effect of selection;

## Discussion

### Proportion and type of heteroplasmy: coding region *vs *D-loop

The analysis of individuals belonging to 46 families of Azorean ancestry (Fig. 1) reveals that in 6.5% of the families there is at least one individual that presents mtDNA heteroplasmy produced by mutation in the analyzed coding region. This value is significantly lower than that obtained by Santos et al. [37] for the D-loop (72.9% – 35 of 48 families) using almost the same set of families (Proportion test: Z = -6.556; p < 0.0001). This difference is still significant (Proportion test: Z = -2.009; p = 0.0222) if only point heteroplasmy is considered for the D-loop (20.8% – 10 of 48 families).

As expected in the relative absence of selection, coding homopolymers suffer the same mutational mechanism as those in noncoding regions [7]. However, notable differences between the coding fragment under analysis and the D-loop were also observed in the behavior of the poly-C tracts located in the *ND1 *gene (Table 4). The poly-C tracts of both hypervariable regions I and II are known to have high insertion/deletion rates, thus originating length heteroplasmy [46]. This type of heteroplasmy is represented by multiple populations of mtDNA containing poly-C stretches of various lengths [47]. Santos et al. [37] reported length heteroplasmy produced by the insertion of cytosine residues in the poly-C tract of HVRI and HVRII respectively in 22.92% and 54.16% of the families studied (Table 4). It is accepted that a general mechanism for generating such variability would be replication slippage [48]; in fact, mispairing during replication of repeated sequences, including homopolymers has been commonly suggested to explain length variation in DNA of prokaryotic, eukaryotic and cytoplasmic origin. However, no cases of length heteroplasmy were found in the *ND1 *poly-C tracts. Moreover, in a population database of almost ~1500 sequences no instance of insertions or deletions in this region was observed (Table 4). To better address this question, we selected for analysis all the poly-C tracts across the human mtDNA genome that present similar features to that of the hypervariable regions: a minimum of seven cytosine residues that may be or not interrupted by another base. For the 14 regions retained, variation produced by insertion or deletion of cytosine residues were only observed in non-coding regions and in the 12S sub-unit of ribosomal RNA (rRNA) (Table 4). In the 12S rRNA we identified two repetitive regions (Table 4); however, only one is polymorphic. The polymorphic region of 12S rRNA is located in an internal loop of the secondary structure whereas the non-polymorphic is located in a stem; this agrees with the Ruiz-Pesini and Wallace [49] observation that stem nucleotides are under greater selective constraint than loop nucleotides and it seems that the insertion/deletion of cytosine residues located in this loop would not interfere with the secondary structure of the rRNA. Therefore, differences in mutational patterns observed between coding and non-coding regions (or less functionally relevant regions) are likely the result of selection, operating at the most basic levels of organization, the cell or even the mitochondrion.

**Table 4:** Frequency of insertions/deletions in poly-C tracts (minimum of seven cytosine residues that may be or not interrupted by another base) of human mtDNA from a database of 1457 sequences.

Motif	Position	Region	% in/del in database individuals	% heteroplasmic families in empirical studies
CCCCCCCTCCCCC or CCCCCCCTCCCCCC	303–315	D-loop (Hypervariable region II, conserved sequence block 2)	63% (566/897)	54.16% (26/48)^b^
CCCCCGCCC	494–502	D-loop	0% (0/1457)	-
CCACACCCCCAC	797–808	12S ribosomal RNA	0% (0/1457)	-
CACCCCCTCCCC	954–965	12S ribosomal RNA	2.7% (40/1457)	-
CCCCCCTCCCC	3566–3576	NADH Dehydrogenase subunit 1	0% (0/1457)	0% (0/46)
CCCAACCCCC	3580–3589	NADH Dehydrogenase subunit 1	0% (0/1457)	0% (0/46)
CCCCCCACCC	7397–7406	Cytochrome c oxidase subunit I	0% (0/1457)	-
CACCCCCTCTACCCCCTCT	8270–8288	non-coding region	0.3% (4/1457)^a^	-
CCCCTACCCCCC	9526–9537	Cytochrome c oxidase subunit III	0% (0/1457)	-
CCCCCGCCC	10192–10200	NADH dehydrogenase subunit 3	0% (0/1457)	-
CCCCCCTCC	10947–10955	NADH dehydrogenase subunit 4	0% (0/1457)	-
CCCCCGCATCCCCCTTCC	13754–13771	NADH dehydrogenase subunit 5	0% (0/1457)	-
CCTCCCCC	14528–14535	NADH dehydrogenase subunit 6	0% (0/1457)	-
CCCCCTCCCC	16184–16193	D-loop (Hypervariable region I)	24.7% (222/897)	22.92% (11/48)^b^

a. Excluding the polymorphic 9 pb deletion, typical of haplogroup B; Data from Santos et al. [37]

### Factors influencing mutation rate estimation in pedigrees

The mutation rate obtained for the coding region of mtDNA considering only germinal mutations was 0.2334 mutations/site/Myr, the highest value reported so far, and much higher than those obtained by phylogenetic methods (Table 3). Moreover, empirical estimations for the D-loop and for the coding region of mtDNA (see Table 3 and references therein), clearly show that the evolutionary rate was higher for the D-loop, as it was previously demonstrated by other works [31,41].

The possible causes for the discrepancy between phylogenetic and empirical estimations of mutation rate will be discussed further ahead, implicating mainly the process by which heteroplasmy is resolved as well as the effect of sex proportion and selection at the different nested hierarchies of populations covered by the mtDNA molecule (the organelle, the cell, the tissue, the organism, and so on up through natural populations and species) [8].

For the coding region analyzed, confirmed germinal mutations occurred in very stable positions conserved among species. These results reinforce our previous observations for the D-loop [37] that the discrepancy observed in the mutation rate reported in family and phylogenetic studies can not be attributed to the inclusion of somatic mutations in calculations or to the use of families with mtDNA disease, neither to the occurrence of mutations preferentially in hypervariable sites. As it was previously realized by Santos et al. [37], there are other factors that may be considered in the estimation of mutation rate using empirical approaches; mainly, the sex of individuals where new mutations were found (this correction would be the equivalent of considering the effective population size for the coalescent estimation of the mutation rate, N, for the mtDNA [50]), as well as the probability of intra-individual fixation of the new arising mutations (for a detailed explanation see Santos et al. [37]).

The accuracy of the proposed corrections can be ascertain by two different results: 1) if we consider the data of Heyer et al. [36] (only homoplasmic mutations were found and the number of men and women analyzed were similar) and we correct for gender proportion the mutation rate would be 5.85×10^-6^mutations/site/generation, a value similar to that obtained by Santos et al. [37] for the D-loop, after correcting for the sex of transmitters and for the presence of heteroplasmy; 2) the value obtained for the D-loop [37], after correcting for sex and heteroplamy is similar to that reported by Ho et al. [41] using population data (Table 3).

It is important to note that our study addresses a different question to that analyzed by Ho et al. [9,41]. While they are analyzing the discrepancies in the "rate of change" when it is estimated using short or long-term calibration points, our concern is precisely the process inside the rate of change, i.e., the factors responsible for the differences between the mutation and substitution rates. Therefore, in the comparisons of Ho et al. [9,41] the short-term points are represented by populations, where the mutations observed are already fixed at least at the individual level (they do not report any effect derived from heteroplasmic mutations). Moreover, our results support the idea proposed by Emerson [42] that it would seem likely that the time-scale upon which the pedigree rate converges to the evolutionary rate is very much shorter than the timescale which Ho et al. [9] have focused on. Furthermore, we have to consider the higher uncertainty surrounds the estimation of short-term mutation rates than in the estimation of long-term rates [9]. This uncertainty results from the fact that smaller errors in calibration points may have larger consequences in short-term mutation rates and "because as the calibrations move closer to the present, there is a decreasing amount of information in the sequences" [41]. Even when using ancient DNA sequences, as proposed by Ho et al. [41], we should consider that an haplotype observed at any given time could represent a time span of thousands of years, so radiocarbon dates will provide an additional source of error in determining the actual age of the haplotype.

Assuming that the correction for sex and heteroplasmy are important to empirically estimate the evolutionary rate of mtDNA, if we take into account these two factors, and we assume that mutations detected are neutral or nearly neutral (and by consequence that the probability that the new variant will eventually become fixed – homoplasmic – in the individual is approximately the proportion of the new variant) the number of new mutations that can be fixed at the individual level decreased to 0.202 (if only confirmed germinal mutations are included) – 0.3525 (if all the mutation are included) and by consequence the mutation rate would be reduced to 0.0236 mutations/site/Myr – 0.0411 mutations/site/Myr (Table 3). However, our data clearly evidences that some of the mutations detected cannot be considered neutral and by consequence its probability of intra-individual fixation may be smaller than the frequency in heteroplasmic individuals. As referred by Santos et al. [37] a suitable strategy to solve this problem would be to empirically estimate the proportion of intra-individual fixation by observing the number of heteroplasmic transmissions that become fixed for the new variant. In these circumstances, all the factors affecting the segregation of heteroplasmy, including selection, would be averaged and therefore implicitly considered. Furthermore, assuming that selection pressure will be similar at the intra-individual level and the population level, the rate of mutations that reach intra-individual fixation will be similar to the rate of population (and species) fixation. This means that only neutral and mildly deleterious mutations will be fixed at an intra-individual level but not deleterious mutations, as is observed in the majority of mitochondrial diseases in which the mutant type never reaches intra-individual fixation. Selection, however, could still modify the population fixation rate in cases in which a change in environment modifies the selective pressure [4]. Unfortunately, at this time, we cannot empirically estimate the probability of intra-individual fixation and we will try to access this question by indirect approaches.

The new mutation arising in family AV in position 3260 has been associated with the development of maternally inherited myopathy and cardiomyopathy [51,52]; the wild-type A^(3260) ^is normally bound to the U^(3272) ^as part of a 4 bp palindrome that forms the anticodon stem of the *tRNA*^*Leu*(UUR) ^cloverleaf, the G^(3260) ^mutation creates a non-standard base pair with the corresponding U^(3272)^. The A-U pair is highly conserved through mammalian species [29] and it is probable that the G^(3260) ^affects the stability of the tRNA anticodon stem [51]. The biochemical analysis of oxygen consumption in transformant cybrids suggested that for the G^(3260) ^mutation the threshold for detectable impairment of mitochondrial respiration is higher than 60% mutant mtDNA [52]. Moreover, all symptomatic patients described by Zeviani et al. [51] had a percentage of mutated mitotypes in muscle of at least 87%, whereas one unaffected individual had a percentage of mutant molecules of around 78%. These observations are consistent with our finding that the individuals used in this study are healthy individuals, since the percentages of mutant mtDNA detected in the three individuals of AV family are lower than 20%. The challenge is now to predict the fate of this new arising pathogenic mutation; the IV3 man cannot transmit the new mutation; however her sister can have descendents carrying the mutation. Assuming that selection will not act against the G variant, the fate of the mutation will be entirely governed by drift. According to the model proposed by Santos et al. [37] (that allowed us to predict the probability that a variant drift from ~20% of copies to more than 60% in one generation) it is unlikely that the disease will affect the children of IV4. Moreover, even if this mutation can reach fixation at the individual-level in a future generation, it would never reach fixation at the population level, unless an environmental change, or any factor relaxing the selective pressure, reduce its negative effect. Mitochondrial DNA evolution at the population level may be modulated for example by the climatic variables [4] or by the diet of individuals [29].

The non-synonymous A to G mutation at position 3602 was not described so far, both at the population level or associated to pathology. The conservation index of this position is 100% indicating that it has a functional relevance and that probably is not neutral. The problem here is to determine if this mutation has a negative or positive effect. At this time, it is difficult to determine this issue without any functional assay. Furthermore, we cannot predict if weather, or any other factor, will change in the future, changing in turn the effect of the mutation.

Taking together these evidences, the number of mutations that reach fixation at the individual level should range from 0 (if both germinal mutations detected are considered to be deleterious) to 0.02 (if it is assumed that the mutation in position 3602 is in fact neutral). This represents a mutation rate for the coding fragment analyzed ranging between 0 mutations/site/Myr and 0.0023 mutations/site/Myr and similar to that obtained using phylogenetic approaches (Table 3). If we include the mutation in position 3396, the mutation rate for the coding fragment analyzed ranges between 0.0176 mutations/site/Myr and 0.0199 mutations/site/Myr. These value are similar to that values reported by phylogenetic estimations (Table 3).

## Conclusion

Given the importance of mitochondrial function, changes in the mtDNA sequence can have a substantial impact on the fitness of the organelle/cell/individual and may be eliminated at the different levels of organization of mtDNA. Our results are in agreement with this assumption. First of all, major differences were detected in the proportion and type of heteroplasmy found in the coding and non-coding regions of mtDNA. If we assume that mutations occur randomly in mtDNA genome, the present results support the idea that newly arising mutations in the coding region are eliminated probably at the organelle and cell levels, before they reach a detectable frequency in heteroplasmy. Moreover, some of the mutations found in heteroplasmy in the coding region would never reach fixation at the individual level, since they appear to be deleterious, and would be latter eliminated.

Our empirical estimation of mtDNA coding region mutation rate, calculated taking into consideration the sex of individuals carrying new mutations, the probability of intra-individual fixation of mutations present in heteroplasmy and the effect of selection, is similar to that obtained using phylogenetic approaches. Based on our results, the discrepancy previously reported between the human mtDNA coding region mutation rate observed along evolutionary timescales and estimations obtained using family pedigrees can be understood when correcting for the above cited factors.

## Methods

### Genealogies and Samples

The genealogical information and sample databank were previously described in Santos et al. [37]. All the individuals included in the sample bank were informed by members of the research team about the aims and ethical guidelines of the project and signed an informed consent form before the sampling procedure, conducted by mouth scrap. For this work, we selected 406 individuals belonging to 46 independent mtDNA lineages and corresponding to 311 mtDNA transmissions (Fig. 1).

### General Strategy

The analysis of the samples was done following a strategy similar to that used in Santos et al. [37], with 5 main steps:

1) DNA extraction, PCR amplification and sequencing of the region that encompasses the *tRNA*^*Leu*^, *ND1 *and *tRNA*^*Ile *^genes, of the individuals from the most recent generation (represented by filled black squares and circles in Figure 1); 2) Confirmation by a second PCR amplification and sequencing of the individuals that appeared to be heteroplasmic in step 1; 3) DNA extraction, PCR amplification and sequencing of the ancestors of individuals confirmed as heteroplasmic in step 2 (individuals represented by half tone squares and circles in Figure 1). Whenever heteroplasmy was not present in the mother, the paternal mtDNA was analyzed to exclude a possible paternal contribution; 4) To authenticate results an independent DNA extraction, PCR amplification and sequencing were subsequently performed for all individuals showing heteroplasmy; 5) Cloning and sequencing of multiple clones of individuals that presented heteroplasmy and that are relevant to the mutation rate calculation.

### mtDNA analysis

#### DNA extraction, PCR amplification and sequencing

DNA from the buccal cells was extracted using the JETQUICK Blood and Cell DNA Purification Kit (Genomed) according to the manufacturer's specifications. The mtDNA region from position 3120 to 5129 (according to the revised Cambridge Reference Sequence – CRS [43]), was amplified using new designed primers L-MT3120 (5'-CCTGTACGAAAGGACAAGAG-3') and H-MT5129 (5'-GTTGAGTAGTAGGAATGCGGT-3'). The new primers were verified, using the BLAST procedure, to prevent the amplification of nuclear mitochondrial pseudogenes. The PCR reaction program involved an initial 5 min. denaturation step at 94°C followed by 35 cycles of 94°C for 1 min., 57°C for 45 s and 72°C for 2 min., and a final extension step of 7 min. at 72°C. PCR products were purified using the JETQUICK PCR Purification Spin Kit (Genomed).

All samples were fully sequenced between positions 3230 and 4331 (*tRNA*^*Leu*^, *ND1 *and *tRNA*^*Ile *^genes) using four new designed primers, two in the direct direction [L-MT3120 (5'-CCTGTACGAAAGGACAAGAG-3') and L-MT3746 (5'-CCATCATTCTACTATCAACA-3')] and two in the reverse direction [H-MT3889 (5'-GGTTGGTCTCTGCTAGTGTG-3') and H-MT4432 (5'-GGCCCGATAGCTTATTTAG-3')]. Sequence reactions were carried out using the sequencing kit BigDye^® ^Terminator v.3 (Applied Biosystems) according to the manufacturer's specifications and ran in an ABI Prism 3100 sequencer.

#### Cloning

To quantify the proportion of heteroplasmy in samples that appeared to be relevant to mutation rate calculation, a PCR using primers L-MT3120 and H-MT3889 was performed. The PCR products were subsequently cloned into the pCR^®^4-TOPO^® ^vector with the TOPO TA Cloning^® ^Kit for Sequencing (Invitrogen) and multiple clones for each sample were sequenced using the same primers used for PCR.

### Data analysis

#### Detection of heteroplasmy and Sequence alignment

Sequences were analyzed using the Sequencing Analysis 5.2.0 software (Applied Biosystems), considering a value of 2% in the option of Mixed Base Identification. Moreover, al the sequences were visually verified and compare with other from the same run to detect the presence of heteroplasmy. Only sequences with a good intensity and with background almost imperceptible were used.

Subsequently sequences were aligned in relation to the revised CRS [43], using BioEdit [53]. All polymorphic positions were confirmed in sequencing electropherograms. Sequences without ambiguities were obtained between positions 3230–4331.

#### Levels of heteroplasmy

Levels of heteroplasmy were determined as described in Santos et al. [37] using the height of the peaks in the electropherograms and/or the cloning of PCR products.

#### Population Database and Reference Sequences

To calculate the frequency of each variant for a particular nucleotide position, we created a population database of 1457 mtDNA coding region sequences [2,28,54-59]. To infer the ancestral state in a given nucleotide position we used two sequences: a) the reference sequence proposed by Ingman et al. [28] – GeneBank accession number NC_001807 – that originates from an African sequence (GeneBank accession number AF347015); and b) the *Pan troglodytes *mtDNA sequence – GeneBank accession number X93335 [44].

#### Mutation Rate Estimation

The mutation rate was derived from the number of detected mutations per number of "meioses" or transmission events, which is the number of cumulative generations tracing back to the maternal ancestor. When the mutation rate is expressed as mutations per base pair per million years (Myr), the generational time was assumed to be 25 years [37]. The mutation rate (considering substitutions only) was estimated according to different assumptions: 1) all the substitutions that were detected were considered for the mutation rate calculation, 2) only the substitutions for which a germinal origin was evidenced were considered (applied by the majority of authors, such as [31]), 3) only the substitutions present in women for whom there was evidence of a germinal origin were considered (this takes into account that the mutations present in men have the same evolutionary weight of somatic mutations, since they cannot be transmitted to the next generation), and 4) only the substitutions with a germinal origin present in women that would become fixed at the individual level were considered. To perform these estimations we tried to distinguish if a mutation is neutral or not. A mutation was considered deleterious if it had been previously described as correlated with any form of disease. On the other hand, non-neutral mutations may have functional consequences that may be assessed by analyzing the conservation indices. Neutral mutations would have a low conservation index, whereas non-neutral (deleterious or adaptive) mutations would have a high conservation index [4]. Conservation Index was estimated as in Ruiz-Pesini et al. [4], using the same set of species. For neutral mutations the probability of intra-individual fixation of the new variant was considered to be equal to its frequency in the heteroplasmic individual. For non-neutral deleterious mutations the probability of intra-individual fixation was considered to vary between zero and the frequency in the heteroplasmic individual. The 95% confidence intervals for proportions were computed using the program OpenStat2 v1.42 [60].

## Authors' contributions

CS conducted the molecular typing of samples, performed statistical analysis and drafted the manuscript. RM conducted cloning of samples, collaborated in statistical analysis and participated in the drafted of the manuscript. AA and LA collaborated in the molecular typing of samples. MPA provided laboratorial facilities and revised critically the manuscript. ML was responsible for the study coordination and participated in the drafted of the manuscript. All authors read and approved the final version of the manuscript.

## Supplementary Material

Additional file 1 Detailed results of coding Region sequences. The data provided represent the coding region sequences between positions 3230–4331 of the Azorean families analyzed. Data from Santos et al [37], for the D-loop region, are also presented. Click here for file
