# Supplementary material for: Mutation patterns of mtDNA: Empirical inferences for the coding region
Source: BMC Evol Biol. 2008 Jun 2;8:167. doi: 10.1186/1471-2148-8-167 (PMC2438339; doi:10.1186/1471-2148-8-167)
Supplement: Additional file 1 — Detailed results of coding Region sequences. The data provided represent the coding region sequences between positions 3230–4331 of the Azorean families analyzed. Data from Santos et al [37], for the D-loop region, are also presented. [file 1471-2148-8-167-S1.doc]

Table I – Coding Region sequences between positions 3230-4331 (Andrews et al. 1999) of the Azorean families analysed. Data from Santos et al (2005), for the D-loop region, are also presented.

| **Family** | **Samples** | **Coding Region tRNA^Leu^, ND1 and tRNA^Ile^ (3230-4331)** | **D-loop (16024-16569 and 1-400) (Santos et al 2005)** | **Haplogroup** |
| --- | --- | --- | --- | --- |
| **AC_I** | III1, 2, 3, 4, 5 | - | 16519, 152, 263, 311.1 | H or HV, preHV |
| **AC_II** | II3, III1 | - | 16519, 263, 311.1 | H or HV, preHV |
| **AV** | III9, IV1, 2, 5, 6, 7, 8, 9 | - | 16291, 16298, 72, 195, 263, *Het Poly-C 303-309 (303.1, 303.2, 303.3)*, 311.1 | V or pre*V |
|  | IV3, 4 | **Het 3260 A/G** | 16291, 16298, 72, 195, 263, *Het Poly-C 303-309 (303.1, 303.2, 303.3)*, 311.1 |  |
|  | III3 |  | Not Analysed |  |
|  | II1 | - |  |  |
| **BF** | II1,2,3,4,5 | 4216, 4232 | 16069, 16126, 16278, 16366, 16519, 73, 185, 188, 228, 263, 295, *Het Poly-C 303-309 (303.1, 303.2, 303.3)*, 311.1 [462, 489] | J |
| **BM** | III1, 4, IV1, 2 | 3348, 3969 | 16108, 16111, 16172, 16189, *Het Poly-C 16184-16193 (16190.0, 16190.1, 16190.2)*, 16219, 16278, 16519, 73, 152, 251, 263, 311.1 | U6a1 |
| **CAM_I** | II3, IV2, 3, 4, 5 | 3338 | 16224, 16270, 16T, 73, 150, 199, 263, 279, 311.1, [518T] | U5 |
|  | IV1, 6 |  | 16224, 16270, 16T, 73, 150, 199, 263, 279, *Het Poly-C 303-309 (303.0, 303.1)*, 311.1, [518T] |  |
| **CAM_II** | II1, 2 | - | 16114A, 16192, 16256, 16270, 16294, 16526, 73, 195, 263, *Het Poly-C 303-309 (303.1, 303.2)*, 311.1 | U5a1 |
| **CAM_III** | II1 | - | 16239G, 16256, 16519, 152, 263, *Het Poly-C 303-309 (303.0, 303.1, 303.2, 303.3)*, 311.1 | H or HV, preHV |
|  | II2 |  | 16239G, 16256, 16519, 152, 263, *Het Poly-C 303-309 (303.1, 303.2, 303.3)*, 311.1 |  |
| **CAR_I** | III1, V2, 3, 6, 7, VI1 | **-** | 16274, 16294G, 16519, 152, 263, 311.1 |  |
|  | V1 |  | **Het 16189 T/C***, Het Poly-C 16184-16193 (16190.0, 16190.1)*, 16274, 16294G, 16519, 152, 263, 311.1 |  |
|  | IV1 | Not Analysed | 16274, 16294G, 16519, 152, 263, 311.1 |  |
|  | IV2 |  | 16086, 16256, 16270, 16342, 16399 (Only HVRI) | U5a1a |

| **CAR_II** | II3 | - | 16519, 195, 263, *Het Poly-C 303-309 (303.1, 303.2, 303.3)*, 311.1 | H or HV, preHV |
| --- | --- | --- | --- | --- |
|  | III1 |  | 16519, 195, 263, *Het Poly-C 303-309 (303.1, 303.2)*, 311.1 |  |
|  | II4 |  | 16519, **Het 64 T/C**, 195, 263, *Het Poly-C 303-309 (303.1, 303.2)*, 311.1 |  |
|  | I2 | Not Analysed | 16519, 195, 263, *Het Poly-C 303-309 (303.1, 303.2, 303.3)*, 311.1 |  |
|  | I1 |  | 152, 263, 311.1 (Only HVRII) |  |
| **CAR_III** | II1, 3, 4 | - | 16086, 16104, 16183C, 16189, *Het Poly-C 16184-16193 (16190.1, 16190.2)*, 16223, 16271, 16278, 16519, 73, 153, 195, 225, 226, 263, *Het Poly-C 303-309 (303.1, 303.2, 303.3)*, 311.1 | X |
|  | II2 |  | 16086, 16104, 16183C, 16189, *Het Poly-C 16184-16193 (16190.0, 16190.1, 16190.2)*, 16223, 16271, 16278, 16519, 73, 153, 195, 225, 226, 263, *Het Poly-C 303-309 (303.1, 303.2)*, 311.1 |  |
| **CAR_IV** | II1, 2 | - | 16274, 16294G, 16519, 152, 263, 311.1 | H or HV, preHV |
| **CAR_V** | III1, 2, 3, 4, 5, 6, 7, 8 | 4216, 4232 | 16069, 16126, 16278, 16366, 16519, 73, 185, 188, 228, 263, 295, *Het Poly-C 303-309 (303.1, 303.2, 303.3)*, 311.1 [465, 492] | J |
| **CAR_VI** | II1, 2 | - | 16519, 263, 311.1 | H or HV, preHV |
| **CG_I** | II3, III3, 4, 5, IV1 | 3421, 3594, 3666, 3777, 4104 | 16129, 16187, 16189, 16223, 16265C, 16278, 16286A, 16292, 16294, 16311, 16360, 16519, 16527, 73, 152, 182, 186A, 189C, 195, 198, 247, 263, 297, 311.1, 316 | L1c2 |
| **CG_II** | II1, 4 | - | 16298, 72, 195, 263, *Het Poly-C 303-309 (303.1, 303.2, 303.3)*, 311.1 | V or pre*V |
|  | II2 | **Het 3396 T/C** | 16298, 72, 195, 263, *Het Poly-C 303-309 (303.1, 303.2, 303.3)*, 311.1 |  |
|  | II3, 5 | - | 16298, 72, 195, 263, *Het Poly-C 303-309 (303.1, 303.2)*, 311.1 |  |
|  | I2 | - | Not Analysed |  |
|  | I1 | 3421, 3594, 3666, 3777, 4104 | Not Analysed | Not Analysed |
| **CG_III** | II1,4 | - | 16519, 199, 263, *Het Poly-C 303-309 (303.1, 303.2)*, 311.1 | H or HV, preHV |
|  | II2,3 |  | 16519, 199, 263, *Het Poly-C 303-309 (303.1, 303.2, 303.3)*, 311.1 |  |
| **CM** | IV1, 2 | 3992, 4024 | 16265C, 263, *Het Poly-C 303-309 (303.1, 303.2, 303.3)*, 311.1 | H or HV, preHV |
|  | IV3, 5 |  | 16265C, 263, *Het Poly-C 303-309 (303.0, 303.1, 303.2)*, 311.1 |  |
|  | IV4 |  | 16265C, 263, *Het Poly-C 303-309 (303.1, 303.2)*, 311.1 |  |
| **CV** | IV1, 2, 3, 4 | 3420, 3450 | 16124, 16171, 16183C, 16189, *Het Poly-C 16184-16193 (16190.0, 16190.1, 16190.2)*, 16214, 16223, 16278, 16362, 73, 263, *Het Poly-C 303-309 (303.1, 303.2, 303.3)*, 311.1 | L3b |
| **ER** | II1, 2, 3 | 3394, 4216 | 16069, 16126, 16221, 73, 185, 228, 263, 295, 303.1, 311.1 [464, 484, 491] | J |
| **HR_I** | III1, 2, 7, IV1 | **-** | 16192, 16260, 16519, 263, 311.1 | H or HV, preHV |
|  | IV2 |  | **Het 16189 T/C***, Het Poly-C 16184-16193 (16190.0, 16190.1)*, 16192, 16260, 16519, 263, 311.1 |  |
|  | II2, 3, III5 | Not Analysed | **Het 16189 T/C,** *Het Poly-C 16184-16193 (16190.0, 16190.1)*, 16192, 16260, 16519, 263, 311.1 |  |
| **HR_II** | III1 | 3796 | 16519, 93, 263, *Het Poly-C 303-309 (303.1, 303.2, 303.3)*, 311.1 | H or HV, preHV |
|  | III2, IV1, 2, 3 |  | 16519, 93, 263, *Het Poly-C 303-309 (303.1, 303.2)*, 311.1 |  |
| **JA_I** | III9, IV1, 2, 3, 4, 5, 6, 7, 8, 9, 10 | 4216, 4232 | 16069, 16126, 16278, 16366, 16519, 73, 185, 188, 228, 263, 295, *Het Poly-C 303-309 (303.1, 303.2)*, 311.1 [462, 489] | J |
|  | III10 |  | 16069, 16126, 16278, 16366, 16519, 73, 185, 188, 228, **Het 238 A/G**, 263, 295, *Het Poly-C 303-309 (303.1, 303.2)*, 311.1 [462, 489] |  |
| **JA_II** | II3, III2 | - | 16293, 16311, 16519, 146, 195, 263, *Het Poly-C 303-309 (303.1, 303.2, 303.3)*, 311.1 | H or HV, preHV |
|  | III1 |  | 16293, 16311, 16519, 146, 195, 263, *Het Poly-C 303-309 (303.1, 303.2)*, 311.1 |  |
| **JA_III** | II1, 2 | - | 16183C, 16189,  *Het Poly-C 16184-16193 (16190.0, 16190.1, 16190.2)*, 16519, 187T, 263, 311.1 | H or HV, preHV |
| **JL_I** | II1, 2 | - | 16519, 263, *Het Poly-C 303-309 (303.1, 303.2)*, 311.1 [479] | H or HV, preHV |
| **JL_II** | II1, 2 | - | 16519, 263, *Het Poly-C 303-309 (303.1, 303.2)*, 311.1 | H or HV, preHV |
| **JS** | II5, III1, 2 | 3505, **Het 3602 A/G** | 16223, 16292, 16320, 16519, 73, 189, 195, 204, 207, 263, *Het Poly-C 303-309 (303.0, 303.1)*, 311.1 | W |
|  | III3 | 3505 | 16223, 16292, 16320, 16519, 73, 189, 195, 204, 207, 263, *Het Poly-C 303-309 (303.0, 303.1)*, 311.1 |  |
|  | I2, II2, 3 | 3505, **Het 3602 A/G** | Not Analysed |  |
| **MA_I** | III1, IV1, 2 | 4117 | 16519, 263, 311.1 | H or HV, preHV |
| **MA_II** | II1, 2 | - | 16252, 16270, 16342, 16399, 73, 200, 263, 311.1 | U5 |
| **MA_III** | II1, 2 | - | 16519, 152, 263, 311.1 | H or HV, preHV |
| **MBP** | III3, IV1, 5 | - | 16129, 16183C, 16189,  *Het Poly-C 16184-16193 (16190.0, 16190.1, 16190.2)*, 16223, 16249, 16311, 16399, 16454, 73, 195, 263, *Het Poly-C 303-309 (303.1, 303.2, 303.3)* | M1 |
|  | IV2, 3 |  | 16129, 16183C, 16189, *Het Poly-C 16184-16193 (16190.1, 16190.2)*, 16223, 16249, 16311, 16399, 16454, 73, 195, 263, *Het Poly-C 303-309 (303.1, 303.2)* |  |
|  | IV4 |  | 16129, 16183C, 16189, *Het Poly-C 16184-16193 (16190.1, 16190.2)*, 16223, 16249, 16311, 16399, 16454, 73, 195, 263, *Het Poly-C 303-309 (303.0, 303.1, 303.2, 303.3)* |  |
| **ML** | III1 | Not Analysed | 16129, 16183C, 16189, *Het Poly-C 16184-16193 (16190.0, 16190.1, 16190.2)*, 16249, 16311, 16519, 73, 263, *Het Poly-C 303-309 (303.1, 303.2)*, 311.1 [489] | M1 |
|  | III3 |  | 16129, 16183C, 16189,  *Het Poly-C 16184-16193 (16190.0, 16190.1, 16190.2, 16190.3)*, 16249, 16311, 16519, 73, 263, 311.1 [489] |  |
|  | III2 |  | **Het 16086 T/C,** 16129, **Het 16182 C/A or del A**, 16183C, 16189, *Het Poly-C 16184-16193 (16190.-2, 16190.-1, 16190.0, 16190.1, 16190.2, 16190.3, 16190.4, 16190.5)**, 16249, 16311, 16519, 73, 263, *Het Poly-C 303-309 (303.0, 303.1, 303.2, 303.3, 303.4)*, 311.1, [489] |  |
|  | II4 |  | **Het 16086 T/C**, 16129, **Het 16182 C/A or del A**, 16183C, 16189, *Het Poly-C 16184-16193 (16190.-2, 16190.-1, 16190.0, 16190.1, 16190.2, 16190.3, 16190.4, 16190.5)**, 16249, 16311, 16519, 73, 263, 303.3, 311.1, Het Poly-C 303-309 (303.0, 303.1, 303.2, 303.3, *303.4*) [489] |  |
|  | II2 |  | 16129, 16183C, 16189, *Het Poly-C 16184-16193 (16190.-1, 16190.0, 16190.1, 16190.2, 16190.3)*, 16249, 16311, 16519, 73, 263, 303.2, 311.1, Het Poly-C 303-309 (303.1, 303.2, 303.3) [489] |  |

| **MM_I** | II1, III1, 2 | 3434, 4216 | 16069, 16126, 16145, 16231, 16261, 73, 150, 152, 195, 215, 263, 295, 309.1T, 311.1, 319 | J1a |
| --- | --- | --- | --- | --- |
|  | II2 |  | 16069, 16126, 16145, 16231, 16261, **Het Poly G 66-71 delG**, 73, 150, 152, 195, 215, 263, 295, 309.1T, 311.1, 319 |  |
|  | I2 | Not Analysed | 16069, 16126, 16145, 16231, 16261, **Het Poly G 66-71 delG**, 73, 150, 152, 195, 215, 263, 295, 309.1T, 311.1, 319 |  |
| **MM_II** | II1, 2 | 3918 | 16293, 16311, 195, 263, *Het Poly-C 303-309 (303.1, 303.2)*, 311.1 | H or HV, preHV |
| **MN** | III1, 2, 3, 4 | - | 16189, Het Poly-C 16184-16193 (16190.0, 16190.1, 16190.2, 16190.3), 16298, 72, 263, *Het Poly-C 303-309 (303.1, 303.2, 303.3)*, 311.1 | V or pre*V |
| **NP_I** | III1, 2, 3, 4, 5 | 3348 | 16172, 16183C, 16189, *Het Poly-C 16184-16193 (16190.1, 16190.2)*, 16219, 16278, 73, 263, 311.1 | U6a |
| **NP_II** | III8, IV1, 2, 3 | 4216 | 16069, 16126, 16150, 16519, 73, 185, 188, 228, 263, 295, 311.1 [463, 490] | J |
|  | III1 |  | 16069, 16126, **16150 Het T/C**, 16519, 73, 185, 188, 228, 263, 295, 311.1 [463, 490] |  |
|  | II2 | Not Analysed | 16069, 16126, **16150 Het T/C**, 16519, 73, 185, 188, 228, 263, 295, 311.1 [463, 490] |  |
|  | III6 |  | 16069, 16126, 16150, 16519, 73, 185, 188, 228, 263, 295, 311.1 [463, 490] |  |
| **NP_III** | IV4, V1 | 3480 | 16224, 16311, 16519, 73, 195, 263, 311.1 [498] | K |
|  | IV1 |  | 16224, **16309 Het A/G**, 16311, 16519, 73, 195, 263, 315 (1C) [498] |  |
|  | III2 | Not Analysed | 16224, 16311, 16519, 73, 195, 263, 315 (1C) [498] |  |
|  | III1 |  | 16519 (Only HVRI) | H or HV, preHV |
|  | IV7, V2, VI1 | 3480 | 16224, 16311, 16519, 73, **150 Het C/T**, 195, 263, 311.1 [498] | K |
|  | V3 | Not Analysed | 16224, 16311, 16519, 73, **150 Het C/T**, 195, 263, 311.1 [498] |  |
|  | IV6 |  | 16224, 16311, 16519, 73, **150 Het C/T**, 195, 263, 311.1 [498] |  |
|  | III5 |  | 16224, 16311, 16519, 73, **150 Het C/T**, 195, 263, 311.1 [498] |  |
| **NP_IV** | II1, 2, 3 | - | 16140, 16519, 263, *Het Poly-C 303-309 (303.1, 303.2, 303.3)*, 311.1 | H or HV, preHV |
| **NP_V** | II1, 2, 3 | - | 16304, 185, 263, 311.1 [456] | H or HV, preHV |
| **PA_I** | IV1, 2, 3 | - | 16129, 16183C, 16189,  *Het Poly-C 16184-16193 (16190.0, 16190.1, 16190.2)*, 16249, 16311, 16355, 16519, 73, 263, *Het Poly-C 303-309 (303.1, 303.2)*, 311.1 | U1 |
|  | IV4 |  | 16129, 16183C, 16189,  *Het Poly-C 16184-16193 (16190.0, 16190.1, 16190.2)*, 16249, **16264 Het T/C**, 16311, 16355, 16519, 73, 263, *Het Poly-C 303-309 (303.1, 303.2)*, 311.1 |  |
|  | III5 | Not Analysed | 16129, 16183C, 16189,  *Het Poly-C 16184-16193 (16190.0, 16190.1, 16190.2)*, 16249, 16311, 16355, 16519, 73, 263, *Het Poly-C 303-309 (303.1, 303.2)*, 311.1 |  |
|  | III6 |  | 16069, 16126, 16278, 16366, 16519 (Only HVRI) | J |

| **PA_II** | II3 | - | 16519, 263, *Het Poly-C 303-309 (303.1, 303.2)*, 311.1 | H or HV, preHV |
| --- | --- | --- | --- | --- |
|  | II1,2 |  | 16519, **152 Het T/C**, 263, *Het Poly-C 303-309 (303.1, 303.2, 303.3)*, 311.1 |  |
|  | I2 | Not Analysed | 16519, **152 Het T/C**, 263, *Het Poly-C 303-309 (303.1, 303.2)*, 311.1 |  |
| **PP** | III1, 2, 3, 4, 5, 6 | 3594, 4104 | 16223, 16278, 16294, 16309, 16390, 73, 143, 152, 195, 263, *Het Poly-C 303-309 (303.1, 303.2)*, 311.1 | L2a |
| **RUB** | II1,2,3,4,5 | - | 16519, 263, 311.1 | H or HV, preHV |
| **SAO** | III1, 2, 3, 4 | Not analysed | 16201, 16278, 16519, **Het 215 G/A**, 263, 311.1 | H or HV, preHV |
|  | II1 |  | 16201, 16278, 16519, **Het 215 G/A**, 263, 311.1 |  |
|  | I2 |  | 16201, 16278, 16519, **Het 215 G/A**, 263, 311.1 |  |
| **TM** | II7, 10, 11, III1, 2, 3, 4, 5, 6, 7 | 3499 | 16239, 16519, 263, 311.1 | H or HV, preHV |
| **ZH** | III5, 6, 7, IV1, 2, 3, 4, 5, 6, 7, 8 | - | 16519, 152, 263, 311.1 | H or HV, preHV |

Substitutions are transitions unless the base change is explicitly indicated.

* Variants detected by sequencing of multiple clones.
